# Sex-specific genetic influence on thyroid-stimulating hormone and free thyroxine levels, and interactions between measurements: KNHANES 2013–2015

**DOI:** 10.1371/journal.pone.0207446

**Published:** 2018-11-14

**Authors:** Young Ki Lee, Dong Yeob Shin, Hyejung Shin, Eun Jig Lee

**Affiliations:** 1 Division of Endocrinology and Metabolism, Department of Internal Medicine, Yonsei University College of Medicine, Seoul, Republic of Korea; 2 Center for Thyroid Cancer, National Cancer Center, Goyang-si, Gyeonggi-do, Republic of Korea; 3 Biostatistics Collaboration Unit, Medical Research Center, Yonsei University College of Medicine, Seoul, Republic of Korea; Consiglio Nazionale delle Ricerche, ITALY

## Abstract

**Background:**

Although a wide range of genetic influences on thyroid-stimulating hormone (TSH) and free thyroxine (fT4) levels have been reported, sex differences in the genetic influences have not been well described.

**Methods:**

We assessed TSH and fT4 levels in 2,250 subjects without thyroid peroxidase antibody, with data obtained from the Korea National Health and Nutrition Examination Surveys (KNHANES) conducted from 2013 to 2015. Using variance decomposition methods, the variation of TSH and fT4 levels was divided into genetic and environmental components common to both sexes, and to males and females separately. The genetic correlation between TSH and fT4 levels was also assessed in both sexes, and in males and females separately.

**Results:**

Narrow-sense heritability for TSH and fT4 were 54% and 56%, respectively. Sex-specific heritability for TSH levels was significantly higher in females than in males (75% and 41%, respectively; p = 0.037). Heritability for fT4 levels was not significantly different between males and females (62% and 52%, respectively; p = 0.335). TSH and fT4 levels showed a negative genetic correlation in females (ρ_g_ = -0.347, p = 0.040) after regressing out the influences of environmental covariates, but this correlation was not present in males (ρ_g_ = -0.160, p = 0.391).

**Conclusions:**

The genetic influences on individual TSH levels were more prominent in females than in males. In addition, female-specific pleiotropy between TSH and fT4 might be a clue that this stronger genetic influences in females would mainly affect thyroid function per se, rather than other TSH-related factors that do not primarily trigger the negative feedback loop between TSH and fT4.

## Introduction

Serum levels of thyroid-stimulating hormone (TSH) and free thyroxine (fT4) are basic quantitative indicators for evaluating thyroid functions. Thyroid dysfunction is defined as thyroid hormone level that is outside the reference values.[1,2] However, ranges for TSH and fT4 in population are far wider than those in individuals, indicating that many factors could influence determination of individual set points of TSH and fT4 levels.[3]

The importance of genetic influences in determining TSH and fT4 levels has been demonstrated in some twin and family studies.[4–9] However, the magnitude of contributions of genetic components is incompletely understood, as a wide range of heritability is observed for both TSH (32–65%) and fT4 (37–89%) levels. Considering the higher prevalence of thyroid dysfunction or thyroid autoimmunity in females,[1] there is a possibility that sex difference plays a role in this variable heritability. However, many of the previous studies were conducted in single-sex populations,[4,7,9] and sex differences were rarely evaluated in a quantitative manner even in the studies that included both sexes.[5,6,8]

The purposes of the present study were to investigate the heritability of TSH and fT4 levels, as well as evaluate sex differences in heritability and interrelations between traits, based on a large database from the Korea National Health and Nutrition Examination Surveys (KNHANES) conducted from 2013 to 2015.

## Materials and methods

### Study subjects

This study was based on data acquired from KNHANES VI, which was conducted from 2013 to 2015. KNHANES is a nationwide, cross-sectional survey of the Korean population performed by the Division of Chronic Disease Surveillance of the Korea Centers for Disease Control and Prevention (KCDC), which assessed the overall health, as well as nutritional and socioeconomic status, of non-institutionalized civilian population in South Korea. KNHANES participants were selected by a stratified multistage clustered probability sampling design, which has been certified as appropriate for representative statistics by the Korea Department of Statistics. The institutional review board of the KCDC approved the KNHANES survey (IRB approval no.: 2013-07CON-03-4C, 2013-12EXP-03-5C, 2015–01-02-6C).

**Fig 1:**
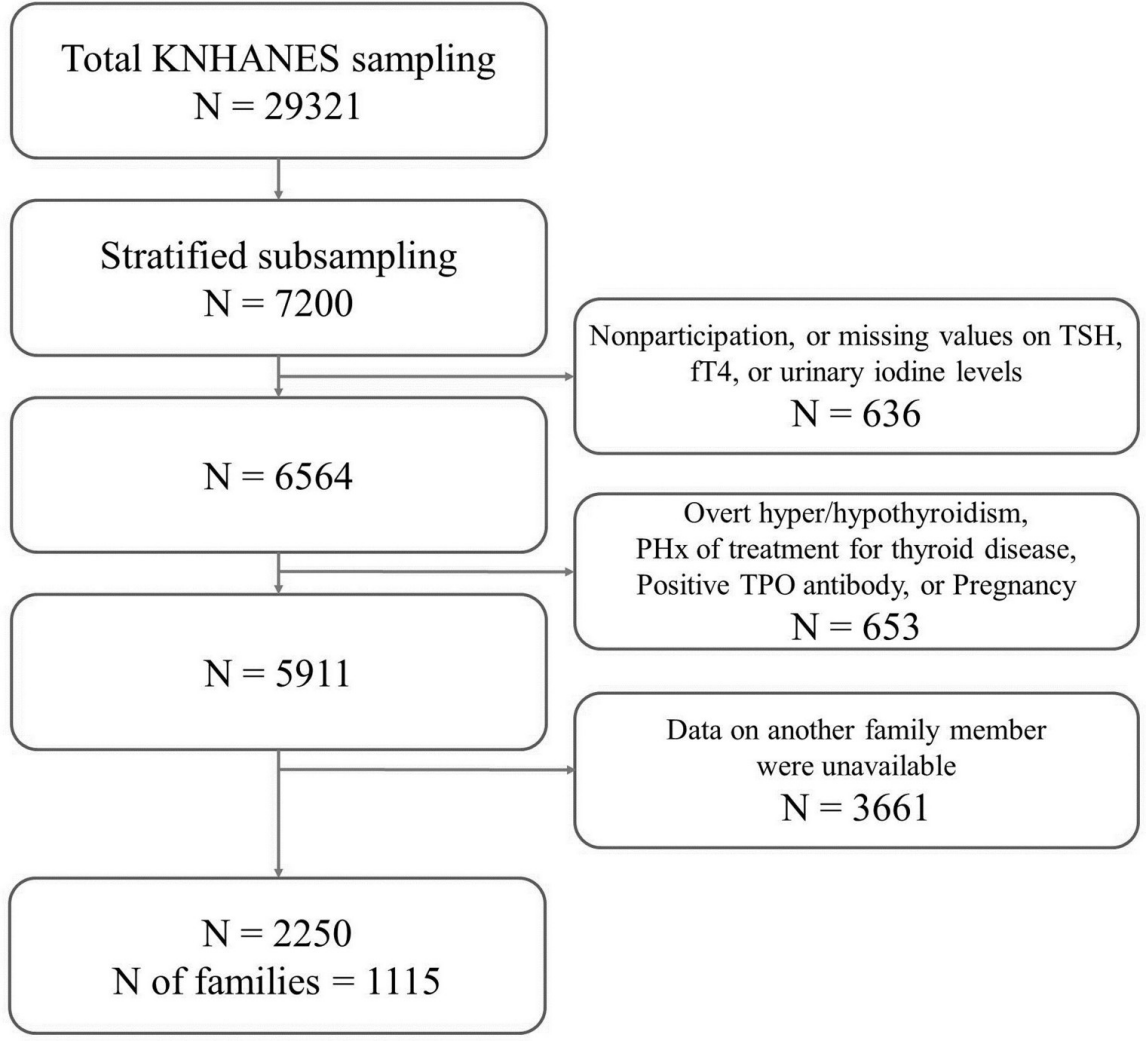
Flowchart of study population selection.

From 2013 to 2015, a total of 29,321 individuals were sampled for the survey. Every year, 2,400 individuals aged 10 years or older were selected by stratified subsampling according to sex and age for measurement of TSH, fT4, thyroid peroxidase antibody (TPOAb), and urinary iodine (UI) levels (Fig 1). Among those participants, 6,564 subjects for whom TSH, fT4, and UI were all measured were screened for the present study. In this subjects, the prevalence of TPOAb positivity was 7.3%, and the prevalence of overt hypothyroidism, subclinical hypothyroidism, overt hyperthyroidism, and subclinical hyperthyroidism was reported to be 0.73%, 3.10%, 0.54%, and 2.98% respectively.[1] Subjects with overt thyroid dysfunction and prior history of treatment for thyroid disease, as well as pregnant women were excluded; in addition, those with positive TPOAb, defined as TPOAb ≥ 34.0 IU/mL (Roche Diagnostics, Mannheim, Germany), were also excluded to minimize the influence of recognizable autoimmune thyroid disease. Families with at least two family members who met the criteria were identified, and in the end, a total of 2,250 subjects (1,201 men and 1,049 women) from 1,115 families were included in the current analyses.

### Definition of euthyroid subgroup

The euthyroid subgroup was defined as subjects with no prior history or family history of thyroid disease and serum TSH and fT4 levels within the reference ranges. Subjects with missing parameters were excluded.

### Laboratory assay

Serum TSH and fT4 levels were measured with an electrochemiluminescence immunoassay (Roche Diagnostics, Mannheim, Germany). TSH was measured using an E-TSH kit (Roche Diagnostics), and its reference interval was considered to be 0.62–6.86 mIU/L, which was the reference interval for serum TSH in Korea based on KNHANES VI data.[1] The fT4 was measured using an E-Free T4 kit (Roche Diagnostics), and the reference interval was 0.89–1.76 ng/mL. UI concentrations were measured with an inductively coupled plasma mass spectrometry device (ICP-MS; Perkin Elmer ICP-MS, Waltham, MA, USA), using an iodine standard (Inorganic Venture, Christiansburg, VA, USA). Urinary creatinine concentration was determined by kinetic colorimetric assay on a Hitachi 7600 automatic analyzer (Hitachi Co., Tokyo, Japan), using CREA reagent (Roche Diagnostics). Urinary iodine (μg):creatinine (g) ratio (UICR) was calculated by dividing urinary iodine by urinary creatinine and then multiplying the result by 100, for adjustment of water excretion rates at the time of spot urine specimen collection.

### Additional variables

Body mass index (BMI), smoking history, menopausal status, past history, as well as family history of thyroid disease were also analyzed in the present study. Height and weight were measured by direct standardized physical examinations conducted at specially equipped mobile examination centers. Data on smoking, menopausal status, prior history, or family history of physician-confirmed thyroid disease were collected through self-report.

### Statistical analyses

Descriptive statistics are expressed as number (%) for categorical variables, and as median value (low quartile, high quartile) for continuous variables. Mann-Whitney U test and Chi-square test were used to compare baseline characteristics of males and females.

Narrow-sense heritability (h^2^) for TSH and fT4, defined by the proportion of phenotypic variance (σ^2^_p_) in a trait that is attributable to additive effects of genes (σ^2^_g_), was estimated by variance components method using SOLAR-Eclipse software package version 8.1.1 (http://solar-eclipse-genetics.org/index.html).[10] Phenotypic variance (σ^2^_p_) was divided into three factors: additive genetic components (A, represents the total sum of additive effects of several alleles), common environmental components (C, represents the influences of environmental factors shared by household members), and unique environmental components (E, represents the influences of environmental factors not shared). The full ACE model was initially fit to the data, and then compared to the nested submodels AE, CE, or E. Significance of each component A or C was assessed by testing the worsening of model fit in the submodels where each component was excluded, with likelihood ratio tests (LRTs). If specific components (A or C) could be excluded from a model without significant worsening of the model fit, the models that did not include the components were regarded to be superior models.

Standard sex-limitation modeling approach was used for quantitative analysis of sex differences in heritability, which were described in the form of genotype-by-sex interactions (G × S interactions).[11,12] Genetic variance σ^2^_g_ was divided into sex-specific genetic variances σ^2^_gM_ and σ^2^_gF_, and environmental variance σ^2^_e_ was divided into sex-specific environmental variances σ^2^_eM_ and σ^2^_eF_. Genetic covariance between same-sex relative pairs was defined as covariance (M1, M2) = 2φ σ^2^_gM_ for males and covariance (F1, F2) = 2φ σ^2^_gF_ for females, where φ was the kinship coefficient. In contrast, the expected genetic covariance between opposite-sex relative pairs was defined as covariance (M, F) = 2φ σ_gM_ σ_gF_ ρ_gMF_, where σ_gM_ and σ_gF_ were sex-specific genetic standard deviations for males and females, and ρ_gMF_ was genetic correlation between the expressions of traits in opposite sexes. In the absence of G × S interactions, genetic correlation should be 100% (ρ_gMF_ = 1), and sex-specific genetic variances should be equal (σ^2^_gM_ = σ^2^_gF_). When the genetic correlation between opposite sexes is less than 100% (ρ_gMF_ < 1), this implies that different subsets of genes may play an important role in the variance in males and females. A difference in the magnitude of the sex-specific genetic standard deviations (σ^2^_gM_ ≠ σ^2^_gF_) indicates that additive genetic influences by the same subset of genes play a larger role in the variance in one sex than in the other. Therefore, the full model, where σ_gM_, σ_gF_, σ_eM_, σ_eF_, ρ_gMF_ were freely estimated, was compared to the nested models that had constraints (ρ_gMF_ = 1, or σ_gM_ = σ_gF_). The LRT statistic follows an asymptotic χ^2^ distribution with 1 degree of freedom when comparing the full model with the nested submodels. However, in the model in which ρ_gMF_ was constrained to 1, the test statistic was distributed as a 50:50 mixture of a χ^2^ distribution and a point mass at zero, because 1 is the upper limit of the ρ_gMF_.[13,14] A significant worsening of model fit in the submodels may serve as evidence for G × S interactions.

We conducted bivariate quantitative genetic analyses to determine whether TSH and fT4 levels were influenced by common genes (pleiotropy).[15,16] Maximum likelihood estimates of both additive genetic (ρ_g_) and environmental (ρ_e_) correlations between TSH and fT4 levels were obtained. Phenotypic correlation (ρ_p_) between TSH and fT4 levels for explaining dependence between relatives was calculated as follows: $\rho_{\text{p}}=\rho_{\text{g}}\sqrt{{\text{h}}^{2}{}_{1}}\sqrt{{\text{h}}^{2}{}_{2}}+\rho_{\text{e}}\sqrt{\left(1-{\text{h}}^{2}{}_{1}\right)}\sqrt{\left(1-{\text{h}}^{2}{}_{2}\right)}$_._

In this equation, h^2^_1_ and h^2^_2_ represent heritability of TSH and fT4 levels, respectively. Significance of each correlation estimate was determined by comparing the likelihood of the model in which ρ_g,_ ρ_e,_, or ρ_p_ is estimated to that of the model where ρ_g,_ ρ_e,_, or ρ_p_ is constrained to zero. Significant genetic correlations (ρ_g_) indicated that common set of genes can influence both traits. Known environmental covariates were adjusted for more sensitive detection of the pleiotropy. In order to identify sex differences in pleiotropy, bivariate analysis was performed in males and females, both simultaneously and separately.

All tests were two-sided, and p-value < 0.05 was considered statistically significant. Statistical analyses, other than genetic analyses, were carried out using SPSS version 20.0 for Windows (SPSS Inc., Chicago, IL, USA).

## Results

Demographic characteristics and thyroid hormone profiles are shown in Table 1. Median age of subjects was 36 years, and median TSH and fT4 levels were 2.30 mIU/L and 1.24 ng/dL, respectively. Males were characterized by lower age, prevalence of family history of thyroid disease, and TSH levels, in addition to higher levels of BMI, prevalence of current smoking, UICR, and fT4 levels compared to females.

**Table 1:** Demographic characteristics and descriptive statistics of study variables.

	Total (n = 2250)	Male (n = 1201)	Female (n = 1049)	P-value
Age (years)	36 (21, 50)	35 (20, 50)	38 (21, 49)	0.523^a^
BMI (kg/m^2^)	22.8 (20.6, 25.4)	23.6 (21.3, 26.0)	22.1 (19.8, 24.4)	< 0.001^a^
Smoking, n (%)				< 0.001^b^
Current	436 (19.4)	376 (31.3)	60 (5.7)	
Former/never	1623 (72.1)	708 (59.0)	915 (87.2)	
Missing	91 (4.0)	65 (5.4)	26 (2.5)	
Not indicated (age < 12)	100 (4.4)	52 (4.3)	48 (4.6)	
FHx of thyroid disease				0.006^**b**^
Yes	94 (4.2)	37 (3.1)	57 (5.4)	
No	2038 (90.6)	1095 (91.2)	943 (89.9)	
Missing or incomplete	118 (5.2)	69 (5.7)	49 (4.7)	
Menopause (female only)				N/A
Yes			236 (22.5)	
No			767 (73.1)	
Missing or unknown			46 (4.4)	
UICR (μg/mL)	181.2 (96.7, 430.8)	164.1 (87.7, 365.3)	205.0 (107.8, 480.5)	< 0.001^a^
TSH (mIU/L)	2.30 (1.60, 3.25)	2.21 (1.55, 3.17)	2.37 (1.68, 3.44)	< 0.001^a^
fT4 (ng/dL)	1.24 (1.14, 1.36)	1.29 (1.18, 1.41)	1.20 (1.10, 1.30)	< 0.001^a^

Abbreviation: BMI, Body mass index; FHx, family history; UICR, Urinary iodine/creatinine ratio; N/A, not assessed.

Values are presented as median (low quartile, high quartile) or number (%).

Unknown menopausal status indicates subjects with hysterectomy or intrauterine devices.

^a^ Mann-Whitney *U* test was used for comparing males and females.

^**b**^ Chi-square test was used for comparing males and females with valid values.

### Heritability

We first used ACE model with all three components, and then tested that model against AE model in which the common environmental component (C) was removed (S1 Table). Common environmental components could be removed from ACE model for TSH and fT4 levels without significant deterioration of model fit (p = 0.056 and 0.095, respectively). Therefore, we rejected ACE models and adopted AE model as the final model. Final heritability estimates are listed in Table 2. Sex-specific heritability estimates were calculated separately in male and female groups. Overall narrow-sense heritability estimates for TSH and fT4 were 54% and 56%, respectively. Males showed higher heritability estimate for fT4 (62%) than did females (52%), while females showed higher heritability estimate for TSH (75%) than did males (41%). All heritability estimates were significant (all p-values were < 0.01).

**Table 2:** Narrow sense heritability of TSH and fT4.

	Heritability (*h*^*2*^)	P-value
TSH		
Total	0.54 ± 0.06	< 0.001
Male	0.41 ± 0.10	< 0.001
Female	0.75 ± 0.12	< 0.001
fT4		
Total	0.56 ± 0.06	< 0.001
Male	0.62 ± 0.10	< 0.001
Female	0.52 ± 0.14	< 0.001

TSH and fT4 values were normalized by rank-based inverse normal transformations. All estimates were adjusted for age, age^2^, and sex using a stepwise (forward and backward) procedure. Heritability was described as estimates ± standard errors.

**Fig 2:**
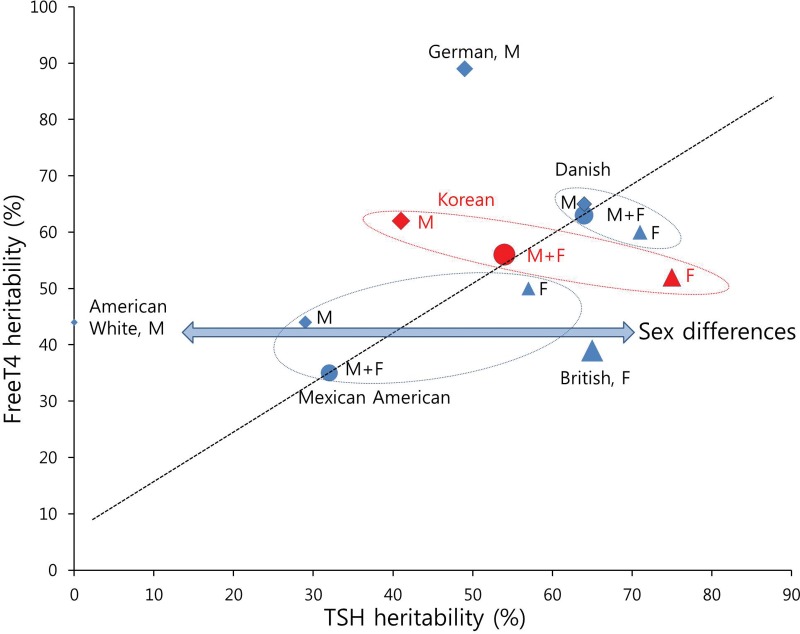
Heritability estimates for TSH and fT4 levels shown in literature and in our study. Overall and sex specific heritability estimates for TSH and fT4 levels from our study and literature[4–9] were plotted. Rhombic markers (M) are male, triangular markers (F) are female, and circle markers (M+F) are male and female. Size of markers represents the number of participants. Ellipse consisting of dotted line indicates a study of the same subject group, or a single study. Region marked “Korean” refers to estimates from this study. Heritability of TSH and fT4 levels shows sex differences (double arrow), as well as proportional tendency (dotted line).

To understand the pattern of the distribution of heritability estimates, we plotted the estimates from our study and from literature in a graph (Fig 2).[4–9] Our heritability estimates were located midway between the values reported in the literature. Visually, heritability of TSH and fT4 showed a tendency to be proportional, as expected from close physiological correlations of the two traits. Heritability estimates from Caucasoid populations (British[4], Danish[5,8], and German[9]) tended to be higher than those from Mongoloid populations (Korean [our study], and Mexican American[6]), suggesting that there might be ethnic differences in heritability of TSH and fT4 levels. In addition, heritability estimates of TSH for female were higher than those for male, when the population studied was the same.

### Genotype by sex interaction

Results of assessment for G × S interactions on variation of TSH and fT4 levels are shown in Table 3. The genetic correlations between males and females were not significantly different from one for either TSH or fT4 levels (p values = 0.478 and 0.455, respectively). However, genetic standard deviation of TSH levels was significantly different between sexes (p-value = 0.037). This finding indicates that the magnitude of genetic influence by the same subset of genes significantly differs between sexes for TSH levels. Statistical significance was intensified after adjustment for environmental covariates (p-value = 0.025). For fT4 levels, genetic standard deviation was not significantly different between sexes.

**Table 3:** Genotype by sex interaction on variance of TSH and fT4 levels.

Traits	Adjustment for environmental covariates	Full model	Nested models					
			ρ_gMF_ = 1			^a^σ_gM_ = σ_gF_		
		-2 log-Likelihood	-2 log-Likelihood	χ2	P-value	-2 log-Likelihood	χ2	P-value
TSH	No^**b**^	2116.24	2116.25	0.00	0.478	**2120.59**	**4.35**	**0.037**
	Yes^**c**^	1848.10	1848.10	0.00	0.500	**1853.13**	**5.03**	**0.025**
Ft4	No^**b**^	1867.06	1867.07	0.01	0.455	1867.99	0.93	0.335
	Yes^**c**^	1587.16	1587.16	0.00	0.500	1588.00	0.84	0.359

Abbreviation: ρ_gMF_, genetic correlation between males and females; σ_gM_, genetic standard deviation in males; σ_gF_, genetic standard deviation in females.

^a^Rejection of null hypothesis (p value < 0.05) indicates that the magnitude of genetic effect differed between sexes.

^**b**^Adjusted for age, age^2^, and sex only.

^**c**^Adjusted for age, sex, BMI, smoking status, log-transformed urinary iodine/creatinine ratio, and menopausal status (males were regarded not to have menopausal status); only subjects without any missing values of covariates were included (n = 2028).

Statistically significant (p < 0.05) values are in boldface.

### Pleiotropy

We conducted bivariate genetic analysis with age, sex, BMI, smoking status, log-transformed UICR, and menopausal status (in females) as covariates (Table 4). Genetic correlation between TSH and fT4 levels was not significant in males (ρ_g_ = -0.160, p = 0.391), while a significant negative genetic correlation between TSH and fT4 levels was observed in females (ρ_g_ = -0.347, p = 0.040).

**Table 4:** Genetic, environmental, and phenotypic correlations between TSH and fT4 levels.

	ρ_g_	ρ_e_	ρ_p_
Total	-0.150 P = 0.103	-0.056 P = 0.619	**-0.109** P < 0.001
Male	-0.160 P = 0.391	-0.083 P = 0.610	**-0.116** P < 0.001
Female	**-0.347** P = 0.040	0.367 P = 0.262	**-0.114** P < 0.001

Abbreviation: ρ_g_, genetic correlation; ρ_e_, environmental correlation; ρ_p_, phenotypic correlation.

Estimates were adjusted for age, sex, BMI, smoking status, log-transformed urinary iodine/creatinine ratio, and menopausal status (males were regarded not to have menopausal status); only subjects without any missing values of covariates were included (n = 2028).

Statistically significant (p < 0.05) values are in boldface.

### Analyses in the euthyroid subgroup

We additionally evaluated heritability, G × S interactions, and pleiotropy in the more rigorously-selected euthyroid subgroup without family history of thyroid disease (n = 1838). Although females still showed higher heritability estimate for TSH (66%) than did males (37%), the absolute difference between the sexes (29% point) was slightly reduced compared to the result from main analyses (34% point) (S2 Table). In addition, the statistical significance of G × S interactions disappeared (S3 Table; the p-value for equivalent genetic standard deviation [σ^2^_gM_ = σ^2^_gF_] = 0.217). However, female-specific negative genetic correlation between TSH and fT4 levels was still significant (S4 Table; p-value = 0.018).

## Discussion

In the present study, we investigated the effect of genetic factors on fT4 and TSH levels, as well as their sex differences, in families without evidence of thyroid autoimmunity by using a well-characterized, national population-based survey database.

The sex differences in narrow sense heritability were demonstrated for several quantitative traits, and females often had larger heritability due to the greater genetic influence from the same subset of genes.[17] In addition, clinical sex differences in thyroid function[1,18] suggested that sex differences may also exist in genetic influences on thyroid function. However, the sex differences in genetic influence on thyroid function have not been sufficiently evaluated in previous family and twin studies. Samollow et al. reported that females showed higher heritability for TSH than males (57% vs. 29%, respectively) using data from a group of 1,011 Mexican Americans, but quantitative comparison between the sexes was not performed.[6] In another study by Hansen et al. using data from 1,380 Danish twins, heritability estimates for TSH were slightly higher in females than in males (71% vs. 64%, respectively), but this sex difference was not statistically significant.[8] The sex differences have also not been fully elucidated by genome-wide association studies (GWAS), which has developed significantly over the last decade. Porcu et al. performed sex-specific meta-analyses of GWAS in 26,420 (for TSH) and 17,520 (for fT4) individuals, but sex differences were only evident for small subsets of genes (PDE8B, PDE10A, and MAF/LOC440389 for TSH; and NETO1/FBXO15 and LPCAT2/CAPNS2 for fT4).[19] Moreover, sex-specific effect sizes on TSH at the variants were larger in males than in females. In the present study, we observed high inheritance of both TSH (54%) and fT4 (56%) levels, with higher heritability for TSH levels in females than in males (75% vs. 41%), which is in line with the results from other traits that the genetic variance is typically larger in females.[17] Such sex difference could be confirmed to be significant through further sex-limitation model analyses for TSH (significant G × S interactions). Since proven single nucleotide polymorphisms (SNPs) to date have been able to account for less than 10% of the variance in TSH,[19,20] our results suggest that sex differences in genetic influence on TSH might be hidden in the 'missing heritability', which has not been revealed in GWAS to date.

In general, the changes which primary affect thyroid function per se induce simultaneous shift of fT4 and TSH levels in the opposite direction by a feedback mechanism, in contrast to the changes which primary affect other thyroid hormone-related components such as the hypothalamus-pituitary functions.[21] From this perspective, genetic factors related to changes in the thyroid function itself could be observed in the form of a negative genetic correlation (pleiotropy) between TSH and fT4 levels. Interestingly, significant negative genetic correlation was observed in females after adjustment for significant environmental covariates. In contrast, genetic correlation was not significant in males. This finding could be a clue that the observed higher heritability for TSH levels in females may be associated with thyroid function per se, rather than with other components including the hypothalamus-pituitary functions. These results could be partially different from the report from Hansen et al., where the genetic correlation between TSH and fT4 levels was not significant; however, sex-specific genetic correlation was not evaluated in the study.[8] In the large meta-analyses of GWAS by Porcu et al., no individual SNPs showed negative genetic correlation between TSH and fT4, and only a variant at the LHX3 locus, which has a role in pituitary development, showed significant positive genetic correlation between the two traits.[19] However, the power to detect pleiotropy was less than 9%, and the subsequent study showed that there was evidence of shared genetic pathways between TSH and fT4 based on the polygenic score analysis.[20] Therefore, larger datasets would be required to more clarify the characteristics of the pleiotropy between TSH and fT4 and its sex differences at the individual SNP level.

To further characterize the heritability, we compared the results from the more rigorously-selected euthyroid subgroup with the results of the main analyzes, and found that the heritability for TSH was reduced and the sex difference was less evident. This might suggest that the genetic predisposition associated with TSH levels outside the reference range partially contribute to the high heritability for TSH and its sex differences. Although we excluded subjects with positive TPOAb, circulating TPOAb are not always positive in autoimmune thyroid diseases,[22] so the TSH levels outside the reference range could still be associated with autoimmune thyroid diseases negative for TPOAb. Alternatively, the conditions could be associated with rare genetic variants with large effect sizes on TSH levels.[20] Data on the presence of thyroid autoantibodies were limited in GWAS of thyroid function so far.[23] Therefore, it is difficult to estimate which of these plays more major roles from GWAS to date, and further studies including individuals with TSH levels outside the reference range and detailed information on the presence of autoantibodies would be helpful.

To our knowledge, the current study is the first Asian study to evaluate the heritability of TSH and fT4, as well as the first nationwide study on this topic. The heritability or genetic effects of TSH and fT4 could vary for different ethnic groups, given the wide ranges of TSH as well as varying incidence of thyroid dysfunction between different ethnic populations.[18,24,25] Therefore, data from non-Caucasian populations could assist in determining the possible ethnic differences in genetic effects on both TSH and fT4. In addition, the present study is the largest study to date, and includes both male and female subjects, so sex differences in heritability could be well assessed. Furthermore, what makes this study more unique and valuable is that it revealed the presence of shared genetic influences between TSH and fT4 levels in females without TPOAb, although not in males.

The present study also had some limitations. Since this was a cross-sectional observational study, the associations observed in this study cannot serve as proof of a causal relationship. Information related to participants’ medical history was collected from self-reports, and a detailed history regarding prescribed medications was not obtained in the survey. Therefore, some subjects taking medications or with comorbidities that could influence thyroid function could be included in this study. Measurements of TSH and fT4 were performed only once for each individual, and no information was provided about the time of day or the season in which TSH and fT4 were measured. Therefore, it is possible that diurnal variation [26] or seasonal changes [27] may have increased the total variation in hormone levels, resulting in smaller heritability estimates. Owing to the lack of data regarding free triiodothyronine (fT3), we could not estimate the heritability of fT3, or assess the interaction between fT3 and TSH or fT4 levels. Since thyroid ultrasound results were not available, we were not able to analyze the size or morphology of thyroid gland, and evaluation of thyroid autoimmunity was dependent on serum TPOAb level.

In conclusion, our findings have confirmed strong genetic influences on thyroid hormone profiles in Korean individuals without TPO antibody, as well as sex differences in the heritability of TSH, and female-specific pleiotropy between TSH and fT4 levels. We believe that these findings will help us to understand the manner in which individual's thyroid hormone profile is determined, as well as its familial interdependence.

## Supporting information

S1 Table Model fitting for TSH and fT4 levels. Abbreviation: A, additive genetic components; C, common environmental components; E, unique environmental components; df; degree of freedom; FHx, family history. TSH and fT4 values were normalized by rank-based inverse normal transformations. All estimates were adjusted for age, age^2^, and sex using a stepwise (forward and backward) procedure. P-values were obtained from likelihood ratio test of AE, CE, or E model compared to ACE model. P-values > 0.05 indicated that removed component did not play a significant role in explaining the data. Estimates were described as mean ± standard errors. Best fitting model is in boldface.
(DOCX)

S2 Table Narrow sense heritability of TSH and fT4 in euthyroid subgroup (n = 1838). TSH and fT4 values were normalized by rank-based inverse normal transformations. All estimates were adjusted for age, age^2^, and sex using a stepwise (forward and backward) procedure. Heritability was described as estimates ± standard errors.
(DOCX)

S3 Table Genotype by sex interaction on variance of TSH and fT4 levels in euthyroid subgroup. Abbreviation: ρ_gMF_, genetic correlation between males and females; σ_gM_, genetic standard deviation in males; σ_gF_, genetic standard deviation in females. ^a^Adjusted for age, age^2^, and sex only. ^**b**^Adjusted for age, sex, BMI, smoking status, log-transformed urinary iodine/creatinine ratio, and menopausal status (males were regarded not to have menopausal status); only subjects without any missing values of covariates were included (n = 1709).
(DOCX)

S4 Table Genetic, environmental, and phenotypic correlations between TSH and fT4 levels in euthyroid subgroup. Abbreviation: ρ_g_, genetic correlation; ρ_e_, environmental correlation;ρ_p_, phenotypic correlation. Estimates were adjusted for age, sex, BMI, smoking status, log-transformed urinary iodine/creatinine ratio, and menopausal status (males were regarded not to have menopausal status); only subjects without any missing values of covariates were included (n = 1709). Statistically significant (p < 0.05) values are in boldface.
(DOCX)
