# Supplementary material for: Sex-specific genetic influence on thyroid-stimulating hormone and free thyroxine levels, and interactions between measurements: KNHANES 2013–2015
Source: PLoS One. 2018 Nov 14;13(11):e0207446. doi: 10.1371/journal.pone.0207446 (PMC6235387; doi:10.1371/journal.pone.0207446)
Supplement: S1 Table — Abbreviation: A, additive genetic components; C, common environmental components; E, unique environmental components; df; degree of freedom; FHx, family history. TSH and fT4 values were normalized by rank-based inverse normal transformations. All estimates were adjusted for age, age2, and sex using a stepwise (forward and backward) procedure. P-values were obtained from likelihood ratio test of AE, CE, or E model compared to ACE model. P-values > 0.05 indicated that removed component did not play a significant role in explaining the data. Estimates were described as mean ± standard errors. Best fitting model is in boldface. (DOCX) [file pone.0207446.s001.docx]

**S1 Table.**

|  | A | C | E | -2 log-Likelihood statistics | χ^2^ | Δdf | P-value |
| --- | --- | --- | --- | --- | --- | --- | --- |
| TSH |  |  |  |  |  |  |  |
| ACE | 0.33±0.15 | 0.11±0.07 | 0.57±0.09 | 2118.1 |  |  |  |
| **AE** | **0.54±0.06** |  | **0.46±0.06** | **2120.6** | **2.5** | **1** | **0.056** |
| CE |  | 0.24±0.03 | 0.76±0.03 | 2123.4 | 5.3 | 1 | 0.010 |
| E |  |  | 1 | 2192.5 | 74.4 | 2 | < 0.001 |
| fT4 |  |  |  |  |  |  |  |
| ACE | 0.40±0.14 | 0.08±0.06 | 0.52±0.09 | 1873.8 |  |  |  |
| **AE** | **0.56±0.06** |  | **0.44±0.06** | **1875.5** | **1.7** | **1** | **0.095** |
| CE |  | 0.23±0.03 | 0.77±0.03 | 1883.2 | 9.4 | 1 | 0.001 |
| E |  |  | 1 | 1945.8 | 72.0 | 2 | < 0.001 |
