# Supplementary material for: Sex-specific genetic influence on thyroid-stimulating hormone and free thyroxine levels, and interactions between measurements: KNHANES 2013–2015
Source: PLoS One. 2018 Nov 14;13(11):e0207446. doi: 10.1371/journal.pone.0207446 (PMC6235387; doi:10.1371/journal.pone.0207446)
Supplement: S2 Table — TSH and fT4 values were normalized by rank-based inverse normal transformations. All estimates were adjusted for age, age2, and sex using a stepwise (forward and backward) procedure. Heritability was described as estimates ± standard errors. (DOCX) [file pone.0207446.s002.docx]

**S2 Table.**

|  | Heritability (*h^2^*) | P-value |
| --- | --- | --- |
| TSH |  |  |
| Total | 0.46 ± 0.07 | <0.001 |
| Male | 0.37 ± 0.13 | 0.003 |
| Female | 0.66 ± 0.14 | <0.001 |
| fT4 |  |  |
| Total | 0.52 ± 0.08 | <0.001 |
| Male | 0.54 ± 0.14 | <0.001 |
| Female | 0.48 ± 0.16 | 0.003 |
