# Supplementary material for: Sex-specific genetic influence on thyroid-stimulating hormone and free thyroxine levels, and interactions between measurements: KNHANES 2013–2015
Source: PLoS One. 2018 Nov 14;13(11):e0207446. doi: 10.1371/journal.pone.0207446 (PMC6235387; doi:10.1371/journal.pone.0207446)
Supplement: S3 Table — Abbreviation: ρgMF, genetic correlation between males and females; σgM, genetic standard deviation in males; σgF, genetic standard deviation in females. aAdjusted for age, age2, and sex only. bAdjusted for age, sex, BMI, smoking status, log-transformed urinary iodine/creatinine ratio, and menopausal status (males were regarded not to have menopausal status); only subjects without any missing values of covariates were included (n = 1709). (DOCX) [file pone.0207446.s003.docx]

**S3 Table.**

| Traits | Adjustment for environmental covariates | Full model | Nested models | | | | | |
| --- | --- | --- | --- | --- | --- | --- | --- | --- |
|  |  |  | ρ_gMF_ = 1 | | | σ_gM_ = σ_gF_ | | |
|  |  | -2 log-Likelihood | -2 log-Likelihood | χ2 | P-value | -2 log-Likelihood | χ2 | P-value |
| TSH | No^a^ | 1752.57 | 1752.65 | 0.07 | 0.393 | 1754.10 | 1.52 | 0.217 |
|  | Yes^b^ | 1589.87 | 1589.92 | 0.04 | 0.417 | 1591.36 | 1.49 | 0.222 |
| Ft4 | No^a^ | 1528.55 | 1528.55 | 0.00 | 0.500 | 1528.82 | 0.27 | 0.605 |
|  | Yes^b^ | 1346.38 | 1346.38 | 0.00 | 0.500 | 1346.43 | 0.05 | 0.823 |
