# Supplementary material for: Sex-specific genetic influence on thyroid-stimulating hormone and free thyroxine levels, and interactions between measurements: KNHANES 2013–2015
Source: PLoS One. 2018 Nov 14;13(11):e0207446. doi: 10.1371/journal.pone.0207446 (PMC6235387; doi:10.1371/journal.pone.0207446)
Supplement: S4 Table — Abbreviation: ρg, genetic correlation; ρe, environmental correlation;ρp, phenotypic correlation. Estimates were adjusted for age, sex, BMI, smoking status, log-transformed urinary iodine/creatinine ratio, and menopausal status (males were regarded not to have menopausal status); only subjects without any missing values of covariates were included (n = 1709). Statistically significant (p < 0.05) values are in boldface. (DOCX) [file pone.0207446.s004.docx]

**S4 Table.**

|  | ρ_g_ | ρ_e_ | ρ_p_ |
| --- | --- | --- | --- |
| Total | **-0.234**  P=0.047 | 0.046  P=0.707 | **-0.102**  P<0.001 |
| Male | -0.089  P=0.732 | -0.126  P=0.185 | **-0.109**  P=0.001 |
| Female | **-0.489**  P=0.018 | 0.507  P=0.094 | **-0.097**  0.006 |
